# An Agent-Based Model of Signal Transduction in Bacterial Chemotaxis

**DOI:** 10.1371/journal.pone.0009454

**Published:** 2010-05-13

**Authors:** Jameson Miller, Miles Parker, Robert B. Bourret, Morgan C. Giddings

**Affiliations:** 1 Department of Computer Science, University of North Carolina, Chapel Hill, North Carolina, United States of America; 2 Bioinformatics & Computational Biology Training Program, University of North Carolina, Chapel Hill, North Carolina, United States of America; 3 Metascape, LLC, Nelson, British Columbia, Canada; 4 Department of Microbiology and Immunology, University of North Carolina, Chapel Hill, North Carolina, United States of America; 5 Department of Biomedical Engineering, University of North Carolina, Chapel Hill, North Carolina, United States of America; National Institutes of Health, United States of America

## Abstract

We report the application of agent-based modeling to examine the signal transduction network and receptor arrays for chemotaxis in *Escherichia coli*, which are responsible for regulating swimming behavior in response to environmental stimuli. Agent-based modeling is a stochastic and bottom-up approach, where individual components of the modeled system are explicitly represented, and bulk properties emerge from their movement and interactions. We present the Chemoscape model: a collection of agents representing both fixed membrane-embedded and mobile cytoplasmic proteins, each governed by a set of rules representing knowledge or hypotheses about their function. When the agents were placed in a simulated cellular space and then allowed to move and interact stochastically, the model exhibited many properties similar to the biological system including adaptation, high signal gain, and wide dynamic range. We found the agent based modeling approach to be both powerful and intuitive for testing hypotheses about biological properties such as self-assembly, the non-linear dynamics that occur through cooperative protein interactions, and non-uniform distributions of proteins in the cell. We applied the model to explore the role of receptor type, geometry and cooperativity in the signal gain and dynamic range of the chemotactic response to environmental stimuli. The model provided substantial qualitative evidence that the dynamic range of chemotactic response can be traced to both the heterogeneity of receptor types present, and the modulation of their cooperativity by their methylation state.

## Introduction

One of the great challenges facing modern biology is the integration of knowledge from diverse experimental sources into a cohesive picture of cellular behavior through time. Computational modeling of cellular pathways plays a key role in this effort, providing the ability to examine and test assumptions, identify areas of incomplete or missing knowledge, explore system parameters, and test hypotheses about system behavior. A common approach is to model biological systems by representing bulk properties and reaction rates using differential equations. Bulk rate models have been both popular and effective for representing metabolic pathways where the populations of proteins can be readily approximated as continuous concentrations.

However, a significant class of modeling problems has spatial and temporal relationships that are cumbersome to represent using continuous equations that assume spatial and temporal homogeneity. Examples include complex boundary conditions such as membranes; self assembly of macromolecular complexes such as ribosomes, viral particles, transcriptional regulators, or receptor fields; and systems that are sensitive to the presence of only a few molecules at a specific location, such as transcription factors and bistable switching [1].

### Agent-Based Modeling

Agent-based modeling (ABM) is an alternative – and potentially complementary – method to these traditional top-down approaches. ABMs differ from other component modeling systems (such as cellular automata) by the continuity of the landscape, the heterogeneity of components, and the stochastic influences in agent motion and interaction. ABM takes a bottom-up approach that represents a system as a collection of agents, components that are programmed to simulate the real-world observed behaviors of the various elements of the system to be modeled. The agents move and interact in a simulated environment referred to as a scape (originally derived from landscape). The scape has a defined geometry approximating spatial features of the target system. Agents are individual objects that represent individual components of the modeled system (e.g. individual proteins in cell). Each agent behaves according to a set of rules representing key features of the modeled system. When placed together in a population, the rule-based interactions of agents with each other and with the modeled environment produce complex, system-wide behaviors that may not be obvious from the individual rules. The emergence of complex interactions from relatively simple rules is termed “emergent behavior.” In agent-based models, one tries to find a simple set of rules, defined for individual class members of a system (proteins in our simulations) that lead to a reproduction of the system's overall dynamics. Models are described and built from the bottom-up, rather than from the top-down.

Agent-based modeling has been applied extensively in the social and economic sciences to represent systems having spatial and temporal dynamics not easily represented by bulk equations (e.g. an equation that represents the aggregate properties of a sand cone in an hourglass, but does not model each individual grain of sand). Examples include vehicular traffic flows that are highly dependent upon spatial configuration of cars, trucks, and road features through time; the socioeconomic patterning of neighborhoods, where for example the use of a single home for illicit drug sales can create a dramatic downward spiral within the surrounding block; and the disappearance of the Anasazi Indians of the Southwestern United States, involving a complex web of interconnected communities and ecologies (for a review of ABM see [2] and the accompanying special issue in the same journal). These ABM approaches differed substantially from traditional social science methodologies such as numerical analysis and statistical inference, however they proved to be effective tools providing novel insights into the modeled systems. ABM is in a similar situation now with respect to the biological sciences, and as an alternative to intracellular networks with bulk rate equations, has only now begun to see application in the modeling of biological systems at the cellular level (e.g. [3]).

### Application of ABM to Systems Biology

The success of ABM in social science applications led us to examine whether it could be used to model biological processes where complex spatial relationships are functionally important. The principles of agent-based modeling are well matched to those operative in cellular biology. A cell consists of a collection of components, such as proteins, protein complexes, membranes, DNA, RNA, metabolites, and so on. Individually, cellular components may operate by simple rules, even though determining those rules experimentally can be difficult. For example, a given protein may interact with other proteins, it may catalyze certain reactions, and it may contain “state” information such as the presence of chemical modifications (e.g. phosphorylation) that modulate its behavior. In many cases, the interactions of system components have important spatial relationships, such as in protein complexes, cooperative interactions, or sub-cellular localization. The global behavior of a functional cell arises from the enormous number of local interactions between relatively simple components, a phenomenon known as emergence [4].

In this study, we examined whether agent-based models could be applied to effectively model intracellular signaling in the chemotaxis network in *Escherichia coli*. We built a system called Chemoscape that models intracellular pathways by representing each protein as an individual agent in the simulation, placed in a simulated cellular scape. Bacterial chemotaxis involves the regulation of swimming behavior to optimize nutrient acquisition and avoid harmful substances in the environment. Chemotaxis presents a number of spatiotemporal modeling challenges, such as the self-assembly of receptor complexes and their subsequent interactions that are involved in the detection of environmental substances with both high sensitivity and high dynamic range; modeling feedback loops between membrane-bound receptors and soluble proteins to regulate sensitivity; and modeling the flagellar motors.

We chose to model the chemotaxis pathway in *E. coli* because there is a large body of quantitative *in vivo* and *in vitro* data available. Furthermore, other groups have extensively modeled chemotaxis, which provides a basis for comparison of the Chemoscape agent-based model we developed. We discuss the strengths and limitations to the agent-based modeling approach, and also discuss the ramifications of Chemoscape with regard to the workings of chemotaxis and receptor fields.

### Bacterial Chemotaxis

There are three major parts to the chemotaxis system in *E. coli*: receptor proteins in the cell membrane, which bind to ligand molecules in the environment and communicate their state to the cell interior; intracellular proteins, which form the logic circuit of the system and decide what kind of response will occur to a given extracellular stimulus; and flagellar motors, which modulate their rotation direction depending upon the output of the intracellular circuit. The rotation of *E. coli* flagellar motors results in two fundamental behaviors: running due to counter-clockwise rotation, and tumbling due to brief reversals in rotation direction [5]. Runs are the default behavior, with intermittent tumbles. This occurs as long as the cell does not detect a change in stimuli from the environment. When there is an increase in attractant (or decrease in repellent), the chemotactic circuit causes the flagellar motors to remain in counter-clockwise rotation, suppressing changes in swimming direction and continuing on a favorable course [6]. If no further changes in attractant/repellent are detected, the system resets itself to the default swimming behavior, in a process called adaptation. The chemotaxis system has several interesting properties, including high sensitivity (the ability to respond to a change of about one part in a thousand in receptor occupancy [7]) and a broad dynamic range (the ability to respond to stimuli and adapt precisely over a million-fold range of background stimulus concentrations [8], [9]).

**Figure 1:**
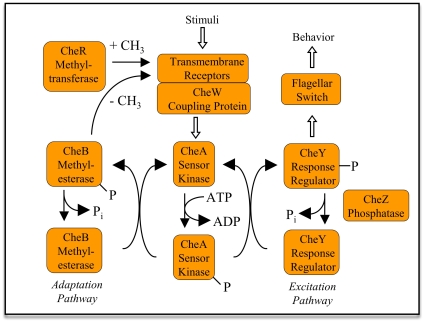
Schematic of the *E. coli* chemotaxis pathway, as described in the Introduction.

The proteins that interact to generate chemotaxis are reviewed in [10]–[12] and illustrated in Figure 1. Transmembrane receptor proteins consist of a periplasmic sensing domain and an intracellular domain that can be variably methylated at specific glutamic acid residues. The number of methyl groups modulates sensitivity of a receptor to ligands, with an increasing number of methyl groups reducing sensitivity to attractant molecules. Receptors are homodimers that appear to complex into trimers of dimers [13], [14]. Receptors have two states, active and inactive, corresponding to whether they are activating the downstream kinase CheA. The inactive state corresponds to an increasing attractant signal. A complex of CheW and CheA proteins binds the intracellular portion of the receptors, and is responsible for translating receptor state to the downstream components of the system. CheW interfaces CheA with receptors. Because CheA is a dimer, the potential exists to connect adjacent receptor clusters through self-assembled CheA−CheW “bridges”. CheA is a histidine kinase that, when receptors are active, autophosphorylates and then serves as a source of phosphoryl groups for the CheB and CheY proteins. Phosphorylated CheY interacts with the flagellar motors to cause clockwise rotation. CheZ is a phosphatase that constantly acts to return CheY molecules to the unphosphorylated state. So the excitation portion of the circuit, driven by CheY state, acts to translate increasing attractant or decreasing repellent into swimming in smooth arcs without tumbles, by shutting off CheY phosphorylation. Likewise, an increase in repellent or decrease in attractant causes an increase in CheY phosphorylation, and hence increased tumbling to change direction.

However, when no further changes in ligand concentration are detected, it is desirable for the cell to resume normal behavior continuing the search for better conditions (e.g. nutrients). The adaptation portion of the circuit, led by CheB, resets the system back to the default swimming behavior. CheB is a methylesterase that is activated upon phosphorylation by CheA and removes methyl groups from the receptors in the active conformation, thus increasing sensitivity to attractants and reducing sensitivity to repellents. By modulating sensitivity this way, CheB drives adaptation by ratcheting the sensitivity up or down depending upon the recent history of receptor activation. CheR is a single-state methyltransferase that constantly works to counter-balance CheB, methylating receptors independently of their state.

The high sensitivity of the chemotaxis information processing system allows state switching of motor proteins in response to the change in state of ligand binding to only a few receptors. Although some of the signal gain is due to CheY-motor interactions [15], most of the gain occurs in the receptor−CheW−CheA complexes [16]. It is a matter of some question how this gain arises, though cooperative interactions between receptor are one explanation [17]. In addition to testing the premise that an ABM can be used to model chemotaxis, we explored receptor cooperativity and methylation, by developing models to explain recent experimental results on the role of receptors in the signal amplification of the system [16]–[18].

## Results

We represented individual membrane bound and soluble proteins as agents, with state information such as conformation or post-translational modification represented as state variables. Agents were placed within a simulated cellular environment laid out as a two-dimensional hexagonal geometry representing discrete cellular locations. Small molecules were represented in bulk because they typically carry no state information, are far greater in number, and have reactions that occur on fast time scales relative to proteins.

We first modeled receptors in both homogenous (single receptor type) and heterogenous (multiple receptor type) populations, with the goal of reproducing key aspects of recent biological experiments illustrating differing effects of distinct receptor types on sensitivity and dynamic range. We then integrated the downstream soluble components into the model, to examine whether the combined system containing fixed and movable agents would exhibit emergent behaviors mimicking those in the biological system, such as chemotactic adaptation. Examples of the model in action are shown in Figure S1 and Movie S1.

The model and source code are available to download from http://bioinfo.unc.edu/Downloads

### Receptor Models

**Figure 2:**
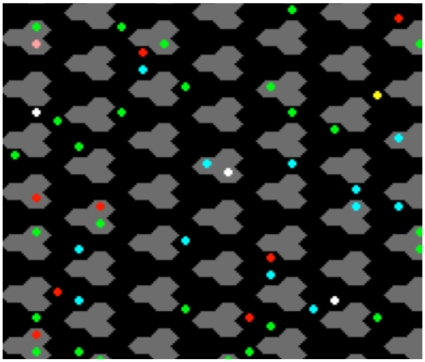
Example simulation. Receptor trimers (gray) arrayed on a hexagonal lattice, with soluble proteins (colored dots) able to move around on top of the array, and to stochastically bind to one another or transfer state information such as phosphorylation/methylation status.

*E. coli* contains at least five receptor types that respond to various ligand stimuli in the environment. Experimental data both *in vitro* and *in vivo* indicate cooperative interactions between these receptors that result in several complex emergent behaviors. We examined receptor interactions by modeling the two major receptor proteins in Chemoscape, Tar and Tsr, which respond to the ligands α-methylaspartate (MeAsp) and serine (Ser), respectively. These receptors play a key role in chemotactic sensing, translating detected ligand concentration into signals used by the downstream apparatus to determine flagellar behavior and cellular motion. *In vivo* chemoreceptors cluster at one pole of the cell in a dense field (receptor patch), and it is believed that the cooperative interactions between trimers of receptor dimers produce the large observed signal gain and dynamic range, although the physical mechanism of interactions is not known. Crystallographic studies suggest that inter-trimer receptors are in contact at their periplasmic tips [19], but it is unclear whether tip contact is the mechanism by which state information is communicated between adjacent receptors. Individual receptor proteins *in vivo*, and the agents representing them in our model, hold state information that includes: conformation, the number of bound methyl groups, and the concentration of interacting ligand molecules. Receptors have a shape that can be approximated by a cylinder [12], so from the two-dimensional perspective of our model they are circular. Geometrically, the maximal packing configuration for circles is hexagonal, so we modeled receptors as fixed agents in hexagonal arrays consisting of trimers of dimers, shown in Figure 2. Recent whole cell electron cryo-tomography experiments demonstrate that bacterial chemoreceptors are packed into hexagonal arrays *in vivo* [20]. Receptors are thought to tightly cluster at one pole of the cell, so the hexagonal model geometry facilitates modeling both tight clustering and the ability of receptors in trimers to have symmetrical contacts with one another, where state information is communicated to model cooperativity. We then modeled the occurrence of inter-trimer interactions when agents are proximal and separated by a single empty lattice cell.

#### Homogeneous receptor arrays

*In vivo*, the conformation of a receptor is determined by its ligand binding state, methylation state, and the conformation of neighboring receptors. Bacteria that are over-expressing a single type of receptor with two methyl groups exhibit high Hill coefficients of about 10 [17]. The Hill coefficient is a parameter that describes the slope of the dose-response curve (equation 4, Materials and Methods). A high Hill coefficient indicates strong cooperativity between adjacent receptors, where the activation/deactivation of one receptor makes it more likely that its neighbors will follow suit.

We modeled cooperativity in homogeneous arrays of Tar receptor agents using MeAsp as the ligand that deactivates them with increasing concentrations. In the absence of an experimentally determined mechanism of cooperativity, we used the experimental value for the Hill coefficient to deduce the cooperativity parameter in simulations with receptor agents in a lattice populated with a single receptor type. To model receptor activity as a function of ligand concentration, cooperative activation effects, and methylation level, we used the free-energy based formulation of Shimizu et al. [21]. This heuristically derived equation simulates the effects on the free energy difference between the active and inactive state of the receptor by bound ligand, methylation level, and cooperativity with neighboring receptors, thus resulting in a ratio of active to inactive receptors that is a direct function of their free energy difference as given by equation 2 (Materials and Methods). Our formulation differs from the original by including a fractional rather than discrete ligand binding term F(l), and for later experiments, a term that introduces a cooperativity dependence on methylation state.

**Figure 3:**
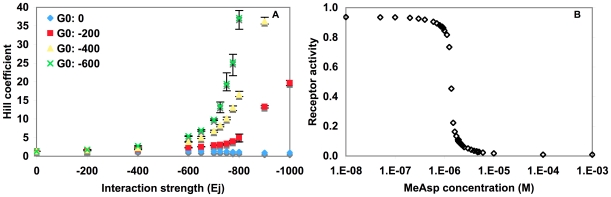
Simulations of a homogeneous receptor population of 1083 Tar receptor dimers. Data points are the mean values from three independent simulations. (**A**) Hill coefficient as a function of interaction strength for four different G_0_ values (blue diamond: G_0_ = 0, red square: G_0_ = −200, yellow triangle: G_0_ = −400, green x: G_0_ = −600). Error bars indicate minimum and maximum Hill coefficients from three independent runs. (**B**) Average Tar receptor activity in a homogenous array, as a function of MeAsp concentration, for an interaction strength of −700 cal mol^−1^ and G_0_ of −600. Fitting the data to the Hill equation resulted in a Hill coefficient of 9.8.

Our simulations modeled an array of 1,083 receptors (Materials and Methods), and were composed of triplicate runs using distinct seeds for the pseudo random number generator. For the initial homogenous receptor array experiment, we examined the influence of the parameters E_j_ (interaction strength) and G_0_ (base activation level) on the Hill coefficient (Figure 3A), because those parameters have not yet been experimentally determined and are affected by model geometry.

While previous models used a *G_0_ = 0* (the ground state, i.e. non-ligand bound receptors are half active), in Chemoscape it was not possible to achieve a Hill coefficient above 3 when using *G_0_ = 0* regardless of the interaction strength *E_j_* (Figure 3A). However, by increasing the ground-state activity level of receptors (decreasing *G_0_*), larger Hill coefficients were obtained as a function of interaction strength. Figure 3B represents the dose-response curve for values *G_0_* of −600 cal mol^−1^ and *E_j_* of −700 cal mol^−1^, giving a Hill coefficient of ∼10. These values were then used as the setting for subsequent simulations.

#### Cooperativity in mixed receptor arrays with a single ligand

In bacteria with multiple receptor types, the Hill coefficient depends on their relative quantities [17]. The more abundant a receptor species (i.e. the closer to a homogeneous receptor population), the higher the Hill coefficient of the response to that receptor's ligand.

**Figure 4:**
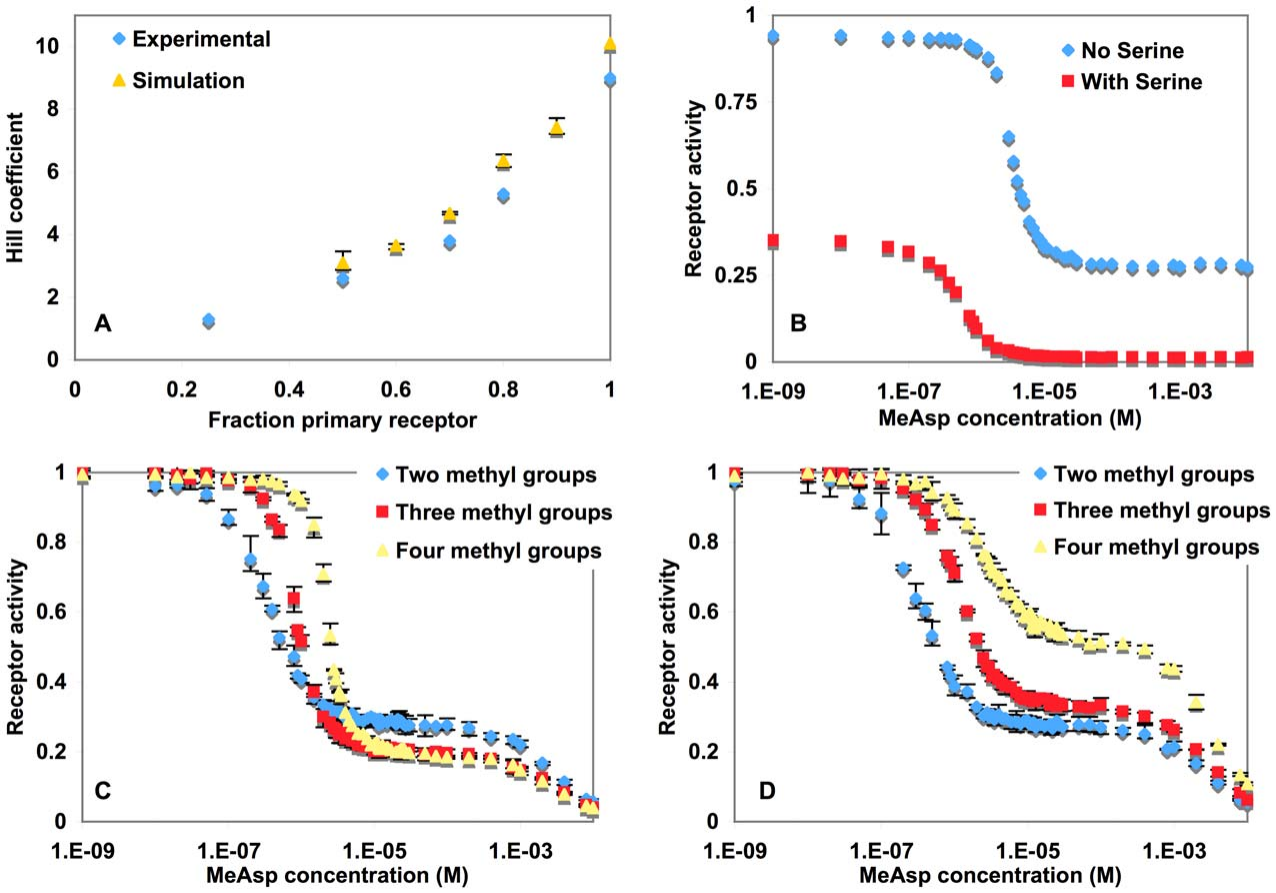
Receptor simulations, each data point derived from the average value of triplicate runs. (**A**) Hill coefficients for the activity of a primary receptor bound by its ligand, as the ratio of primary to secondary receptor was varied, with experimental data from [17]. (**B**) Fractional total receptor activity (Tar+Tsr) as a function of MeAsp concentration, in the absence (♦) or presence (▪) of Ser attractant ligand, which deactivates the Tsr receptors. In the presence of Ser, the baseline activity of the receptors is suppressed to ∼30%, corresponding to the inactivation of the Tsr receptors, and the sensitivity to MeAsp, defined as the ligand concentration at which the activity of the system is one half relative to baseline (K_1/2_), increased ∼6 fold, from 3.3×10^−6^ M to 5.6×10^−7^ M. The Tar receptors have a diminished ability to inhibit the overall activity of the receptor patch, even though they initially account for a larger portion of the overall activity, in agreement with experimental results from [16]. (**C and D**) Plots of combined Tar and Tsr receptor activity versus MeAsp concentration for methylation independent receptor coupling (**C**) and methylation dependent receptor coupling (**D**). In (**C**), as the methylation level increases, there was a greater relative response from Tar binding to MeAsp, contrary to the experimental data from Sourjik and Berg [16], and (**D**) brings the simulation data in line with the experimental data by introducing the term □, which affects receptor cooperativity based on methylation level. (♦: Tar Methylation 2, ▪: Tar Methylation 3, ▴: Tar Methylation 4). Tsr was fixed at methylation level 2 in all experiments.

In our ABM, the separate representation of individual receptors made it straightforward to explicitly represent and model the behavior of a heterogeneous mixture of receptors. We modeled mixed fields of 1083 Tar and Tsr receptor agents, in varying ratios, and tested their response to the ligand MeAsp, the primary deactivator of the Tar receptor, leading to decreased tumbling of the bacteria. We used the parameters G_0_ = −600 cal mol^−1^, with two receptor methylations. The model assumed that MeAsp exclusively bound to Tar, so that Tsr receptors would only be indirectly affected by changes in MeAsp concentration through its cooperative interactions with Tar. The results of the simulations shown in Figure 4A closely matched the experimental observations from Sourjik and Berg [17], showing that as the population becomes more homogenous, the Hill coefficient increases towards that of a purely homogenous population. The good fit between the simulation and the biological system supports the choice of parameters used, but more importantly, shows the ease with which the ABM can represent emergent spatio-temporal behaviors in complex, heterogeneous populations of molecules.

#### Activity in mixed receptor populations with multiple ligands

In bacteria with heterogeneous receptor populations, sensitivity to an attractant ligand that binds one receptor type is increased by the presence of an attractant ligand [17] that binds a different receptor type. The reason for this behavior has not been elucidated, but logically it might be explained by cooperativity between different receptor types. The agent-based model facilitated defining a receptor field composed of a randomly placed mix of two receptor types, and analyzing the effect of cooperativity rules upon the overall receptor response. For the simulations, we used the major receptors Tar and Tsr, which bind to the ligands MeAsp and Ser, respectively. In the simulation, we allowed ligands to bind only their preferred receptor type, in order to elucidate whether cooperative interactions alone might explain the apparent crosstalk between receptors observed in biological experiments.

Using the same receptor patch configuration and parameters as the previous simulation, we modeled the dose-response curves for MeAsp inactivation of the Tar receptor, both in the presence of serine (10^−5^ M, binds Tsr), and absence of serine (Figure 4B). At zero MeAsp concentration, the baseline activity of the receptor population in the presence of Ser was approximately two-thirds lower than in the absence of Ser, due to inactivation of Tsr receptors by the Ser ligand. Notably, there was about a six-fold increase in the sensitivity of the system to the Tar ligand MeAsp in the presence of Ser, as seen by the leftward shift of the response curve. Matching biological experiments, the agent-based receptor models responded at a lower concentration of MeAsp in the presence of Ser than in the absence of Ser, due to cooperative effects. This shows a potential mechanism by which the emergent behavior observed biologically in mixed receptor populations may be explained by straightforward rules governing individual cooperative interactions.

#### Mixed receptor population and methylation

Sourjik and Berg performed experiments with engineered *E. coli* strains having a mixed population of Tar and Tsr receptors, where the methylation state of the Tsr receptors were fixed and the methylation state of the Tar receptors varied by strain [16]. The experiments exposed the strains to varying MeAsp concentrations, which binds and deactivates Tar with a K_d_ that is 10^5^ fold lower than for Tsr. The dose-response of the chemotactic circuit was biphasic, with two distinct concentrations at which receptors became deactivated. It has been postulated that the first phase arises from the primary response of Tar to MeAsp along with a cooperative response of Tsr, followed at a much higher concentration by a secondary response of Tsr directly to the MeAsp ligand. One unexpected result from these experiments was that as the methylation of Tar was increased, the cooperative response of Tsr was reduced.

We examined the properties of the cooperative interactions leading to these response curves, with a Chemoscape model that builds on the previous simulation using a mixed population of Tar and Tsr, in ratio of 1∶2 to mimic the biological experiments. The Tsr methylation level was fixed at two, and Tar methylation level was tested at three different values: two, three, or four. MeAsp was used as the deactivating ligand in the simulation, with stronger binding affinity to Tar and weaker affinity for Tsr.

The first simulations produced the response curves shown in Figure 4C, which mimic the biological results in having a biphasic response, where the first group of receptors deactivate at about 10^−6^–10^−5^M MeAsp due to the primary response of Tar, and the second group deactivates at about 10^−3^M or higher due to the response of the remaining active Tsr receptors. However, the dependence of the plateau level on Tar methylation state was the inverse of that observed in the *in vivo* experiments. One biological explanation for the changes in the dose-response curves at different Tar methylation levels is modulation of receptor cooperativity according to methylation level.

The receptor interaction rules were readily modified in the model to incorporate dependence of receptor cooperativity upon methylation level, given by the term γ in equations 2 and 3 (Materials and Methods). This term modified the coupling strength between receptors to vary inversely with increasing methylation level. Simulations with this modified receptor activity rule were in agreement with experimental results (Figure 4D), with the biphasic response curves as the previous simulation, but showing the distinction that at the higher methylation levels, the modified cooperativity reduced the initial low-dose deactivation response to MeAsp, and proportionally increased the secondary response at much higher MeAsp concentrations. This is because the reduced cooperativity resulted in fewer Tsr receptors being deactivated cooperatively by Tar as the latter respond at low concentrations. Instead, most Tsr receptors were deactivated only as MeAsp concentrations increased to high enough levels to directly deactivate them.

Substantively, this simulation revealed a potential mechanism for the complex emergent behavior noted by Sourjik and Berg, being that cooperativity is inversely dependent on methylation level.

### Complete Chemotaxis Pathway

The soluble components of the chemotaxis pathway play an important role in the excitation and adaptation process, by communicating receptor state to the flagellar motors, and modulating the effect of stimuli on the circuit through changes in receptor methylation state. Previous chemotaxis models have generally represented the soluble components as bulk rate equations. We examined whether the agent-based model could readily represent the soluble chemotactic components, and importantly, whether the fixed receptor and soluble signaling components could be effectively integrated into a single model that displays emergent behavior mimicking key attributes of the biological chemotactic response, such as its wide dynamic range.

We developed a model incorporating both the fixed receptor agent arrays from the previous experiments, and the soluble components CheR, CheB, CheY, and CheZ. The latter were represented as agents that move in a random walk around the model, by choosing an adjacent random location on each iteration. When any of these encounter another agent in an adjacent lattice cell, a rule is activated that allows events such as transfer of a phosphoryl or methyl group from one protein to the other to occur at a certain probability (Materials and Methods).

Two other chemotaxis proteins CheA and CheW were also represented. CheA and CheW presented an interesting case, because in the cell they are initially soluble and free moving, but can bind to form a single CheA−CheW complex with one or two CheW proteins bound to one CheA. Also, CheW can bind to a receptor protein, in which case CheW and any proteins complexed with it become anchored to that receptor. If CheW has not yet partnered with CheA, when a CheA wanders by, they can bind to form a fixed complex. The end result is a series of CheW−CheA−CheW bridges anchored to the receptor array near the cell pole.

The interactions between receptors, CheW and CheA were represented in Chemoscape as follows. When a CheW agent encountered a fixed receptor agent, it could bind and become fixed at that location. Whenever a CheA and CheW (whether fixed or movable) encountered one another, they could bind and become a single “CheA−CheW” complex agent that replaced the individual agents. The CheA−CheW complex could also bind a second CheW to form the complete CheW−CheA−CheW complex. When simulations were begun, all CheA and CheW agents were soluble and free moving, then stochastically self-assembled as a bridge network on the receptor lattice. This macromolecular self-assembly process is a natural consequence of a few appropriate interaction rules in the agent-based model.

The autophosphorylation activity of the CheA agent then depended on the activity of the one or two receptors it was connected to through CheW agents. In turn, soluble CheY agents were phosphorylated by CheA when the two had an encounter, where CheY phosphorylation served as the readout of the system's activation state.

Biologically, the phosphorylation of CheY determines flagellar motor behavior (not represented in the model). Following a stimulus that causes a change in the phosphorylation of the CheY population, adaptation serves to return the system to its baseline state when there is no further change detected in the environment. There is an experimentally observed asymmetry in the adaptive response such that the adaptation to attractant withdrawal occurs more quickly than to attractant addition [22].

We performed simulations to examine whether our model, combining both fixed and movable agents, could readily reproduce the asymmetrical excitation and adaptation response. For the soluble (movable) agents involved with the adaptation response, CheB and CheR, a set of parameters were chosen that resulted in a baseline receptor activity and methylation level with enough range to respond to both positive and negative stimuli. In order for the asymmetrical response to occur in receptor activity (and hence CheY phosphorylation), the relative rate of CheB demethylation had to be larger than the rate of CheR methylation, which is consistent with *in vivo* observations [23]. The larger the difference between these two rates, the larger the asymmetry in the response. The difference in methylation and demethylation rates also influenced the baseline level of receptor activity, i.e. the larger the CheB demethylation rate, the lower the baseline receptor activity, with the opposite effect for increasing CheR rate.

Other parameters necessary for the model included CheA autophosphorylation probability, the probability that phosphorylated CheA would phosphorylate unphosphorylated CheY, and the probability that CheZ would dephosphorylate CheY. Because these values were not known *a priori*, they were set such that a response to both positive and negative stimuli could be observed in levels of phosphorylated CheA and CheY. Setting the CheA autophosphorylation rate too low or too high resulted in CheY phosphorylation levels that responded poorly to stimuli.

**Figure 5:**
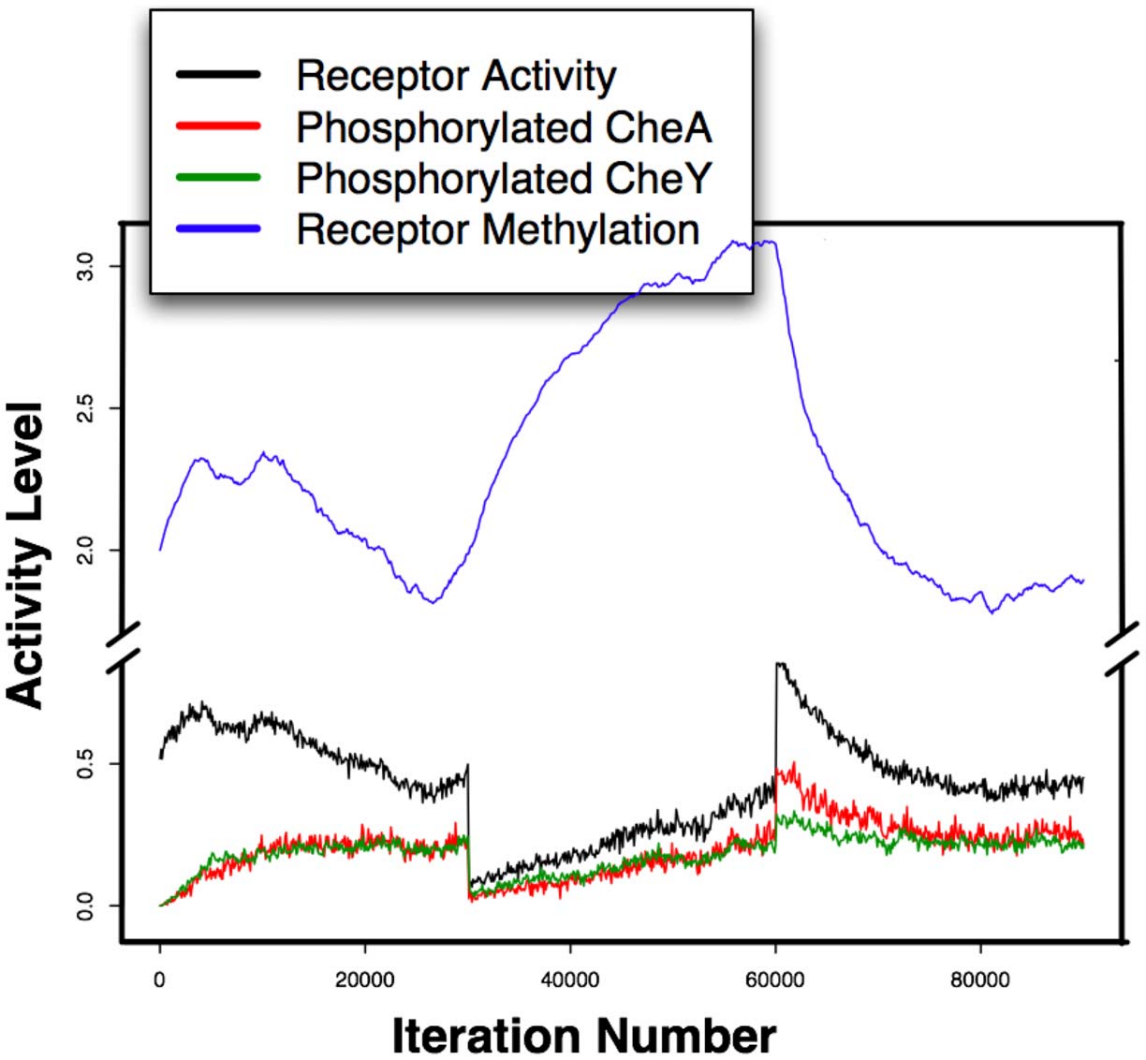
Graph from a single simulation run of a model with a homogeneous Tar receptor population. This model is showing the excitation and adaptation response of fractional receptor activity (black) and average methylation level (blue) in response to the addition and removal of 4×10^−6^ M MeAsp at iteration number 30,000 and 60,000, respectively.

The resulting response to attractant addition followed by attractant removal for a homogeneous population of Tar receptors is shown in Figure 5, with the simulation parameters shown in Table 1. While these were not the only values at which the system would show an adaptive response, they produced the most biological-like behavior of those tested.

**Table 1:** Reactions and corresponding probabilities implemented in Chemoscape.

Reaction Type	Probability of occurrence
CheW+CheA→CheW−CheA	0.01
Receptor+CheW→Receptor−CheW	0.001
CheW−CheA+CheW→CheW−CheA−CheW	1
α_CheA_	0
β	0.03
CheA_p_+CheB→CheA+CheB_p_	0.25
CheA_p_+CheY→CheA+CheY_p_	1
CheR+Receptor→CheR+Receptor_m+1_	0.05
CheB_p_+Receptor→CheB_p_+Receptor_m−1_	0.15
CheY_p_+CheZ→CheY+CheZ	0.2
CheB_p_→CheB	0.005
CheY_p_→CheY	0.0001

The probabilities determine the chance of the corresponding reaction in cases where it is possible, so with bimolecular reactions, the corresponding agents must first encounter one another in an adjacent cell before a reaction is considered at the given probability level.

One of the most striking observations about the biological chemotaxis pathway is its ability to respond sensitively to ligand addition over a very wide dynamic range of ligand concentrations, of 10^6^ or more. Additional simulations tested three different assumptions about receptor cooperativity and their effect on the dynamic range of response for the model: A) homogeneous receptor populations where coupling strength was independent of methylation levels, B) heterogeneous receptor populations where coupling strength was independent of methylation levels, or C) heterogeneous receptor populations where the coupling strength depended inversely on the methylation level of interacting receptors.

**Figure 6:**
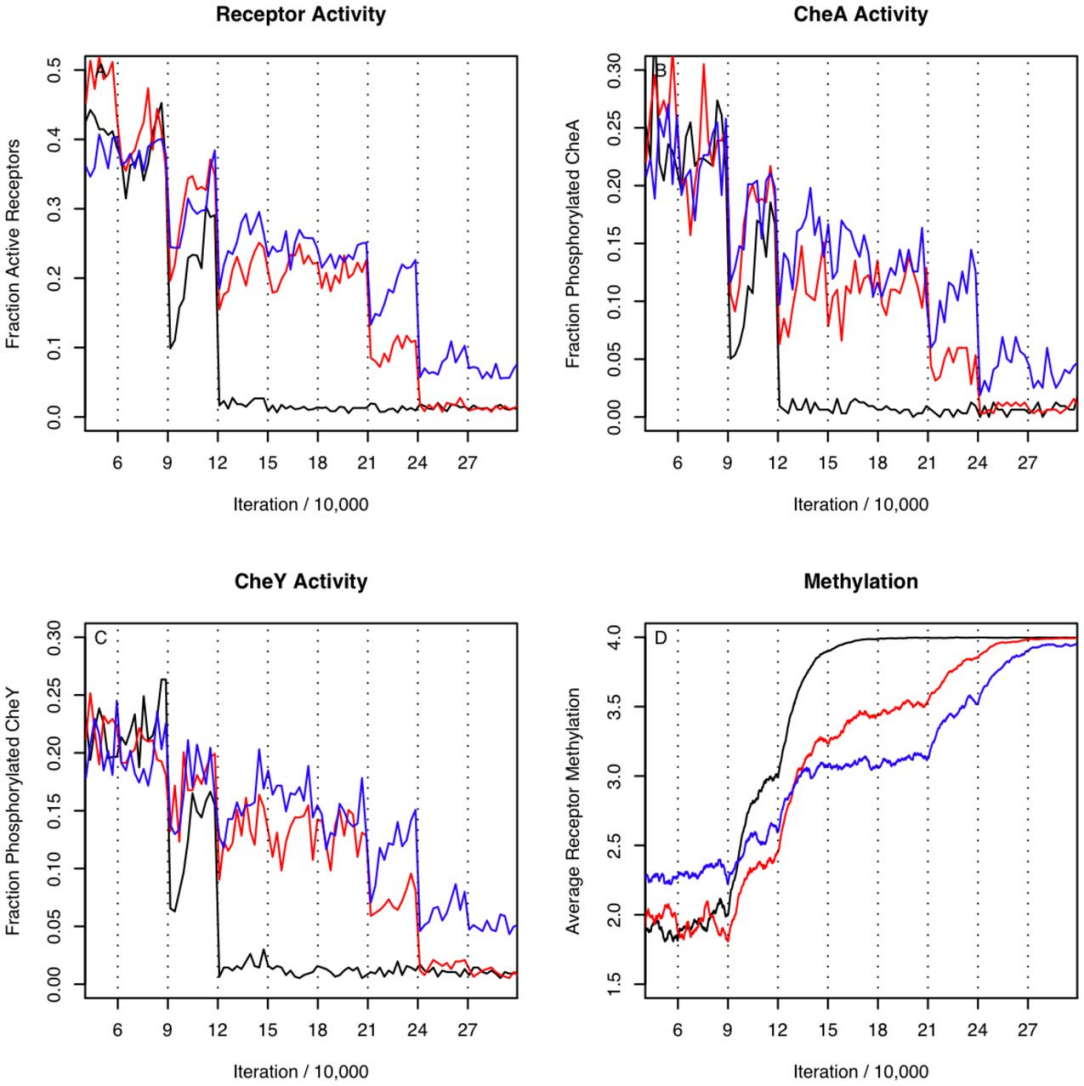
Dynamic range of responses, as a result of ligand addition at varying concentrations. Receptor activity (A), CheA phosphorylation (B), CheY phosphorylation (C) and receptor methylation (D) levels for various ligand increments. Tick marks indicate the iterations at which there was a change in ligand concentration, and were performed at the following iteration numbers and concentrations: (0: 0 M; 30,000: 10^−8^ M; 60,000: 10^−7^ M; 90,000: 10^−6^ M; 120,000: 10^−5^ M; 150,000: 10^−4^ M; 180,000: 10^−3^ M;. 210,000: 10^−2^ M; 240,000: 0.1 M; 270,000: 1 M). The activity of the system reached 0 (saturation) for a homogeneous receptor population (black) at MeAsp concentration of 10^−5^ M, for a heterogeneous population (red): 0.1 M, and for a heterogeneous population (blue) with methylation dependent coupling strength, the activity did not reach 0 during this simulation. The plots for activity levels for receptors, CheA and CheY were smoothed using locally weighted least squares in ‘R’.

The dynamic range was measured as the response of the system as the concentration of MeAsp was increased by an order of magnitude every 30,000 iterations, from 10^−8^ M to 1 M (Figure 6). In the heterogeneous receptor cases, the receptor population was 50% Tar and 50% Tsr. It is interesting to note that while there was no activity of the receptors in the homogeneous model at concentrations greater than 10^−5^ M MeAsp, the heterogeneous model had an extended range of activity compared to the homogeneous model, and the heterogeneous model with methylation dependent coupling strength had an even larger dynamic range. This indicates that the effect noticed by Sourjik and Berg, and represented in our model by varying receptor cooperativity based upon their methylation state, may be largely responsible for the system's emergent property of having a wide dynamic range of response to ligand addition. Our results validate a hypothesis put forward by Bray that cooperativity dependency on receptor methylation may lead to the wide dynamic range observed in the system [24].

## Discussion

### From Rules to Models

In an agent-based model, rules are defined for how we think the individual units of the model behave and interact. In our model, the proteins in the chemotactic circuit are the primary atomic units of the model, and biological knowledge is injected as rules for their behavior. There is nothing explicitly specified in the model for its aggregate or bulk behavior, as those are properties only observed by running the model. For example, the population of phosphorylated CheY protein over time can only be derived by running the model itself, observing how the rules and assumptions made at the protein level affect CheY-P concentration over time. In Chemoscape, we expressed assumptions about how the components were thought to work, and determined whether the resulting bottom-up model matched reality. If performance was off, then something about our assumptions were wrong, and needed consideration or adjustment.

We have sometimes found ourselves subjected to the criticism that agent-based models such as this one are “simplistic”. They are simplistic in the sense that we did not try to formulate a system of equations for the system; instead we let the model do the work directly from the assumptions we expressed about what occurs at the protein level. While this may be a simpler approach in some instances than writing a system of equations, these models can nonetheless be used to represent the complex behaviors of real world systems. The simplicity has a significant benefit of being intuitive and understandable. At the end of molecular biology talks a “model” summary slide for the system studied is usually presented by the speaker. This slide typically shows key molecules and their hypothesized interactions, in a pictorial, bottom-up manner. In developing Chemoscape, the model was developed in much the same way as one of those summary diagrams, readily matching the thinking patterns of experimentalists about the chemotaxis system.

The ability to intuit the model and the basis for its behavior is perhaps the most powerful outcome observed from this chemotactic modeling effort. This ability led to a rapid turnaround time between new hypotheses and their implementation in the model. When the results of the model weren't as expected, we were able to directly understand what went wrong and then improve it.

### Emergent Behaviors

The assumptions we made about protein behaviors, which were coded as rules for single agents, had profound effects on system-wide emergent behavior. For example, one change in the rules governing receptor cooperativity to be based upon methylation state had a major impact upon the dynamic range of the chemotactic system. It was not obvious in advance that making a small modification like this would dramatically enhance dynamic range. This result provided insight into the functioning of the biological system based on single interactions taking place at the biochemical level. The model's insights may help focus subsequent *in vivo* or *in vitro* experiments to further test its results.

From these results, we have come to believe that computational models are most useful at framing biological problems and testing our assumptions about them. If we assume that receptor cooperativity is independent of methylation level, then the receptor patch over-responds to a stimulus, meaning its dynamic range is lessened - regardless of the specific, quantitative parameters used in the model. The quantitative parameters may shift the particular concentration at which the receptors become saturated (all active) - but they don't change the fundamental emergent behavior. The agent-based model facilitated tracing emergent traits back to their roots in the myriad individual biochemical reactions taking place throughout a cell.

### Explicit Spatial Representation and Self-Assembly

Agent-based models explicitly represent spatial relationships between micro-scale system components. The Chemotactic sensing system was a useful example in which to explore the value of this spatially oriented approach. The precise organization of signaling proteins within chemotaxis receptor clusters has not been established, and is an area of active experimental inquiry (e.g. [25], also reviewed in [26]). In this model, we examined a structure in which direct contacts between the periplasmic domains of adjacent trimers of receptor dimers define their primary interactions [19], where the adjacency of trimers defined tertiary interactions.

The model could be readily altered to explore the properties of other receptor array geometries, such as signaling through CheW•CheA•CheW bridges that connect trimers of receptor dimers [27], signaling in linear rows of receptor dimers connected by CheW•CheA•CheA•CheW bridges [28], signaling in two-dimensional lattices formed by direct contact between cytoplasmic domains of receptor dimers [29] or direct contact between CheA molecules attached to different receptors [30]. Another spatial constraint that might be considered are the proposed adaptational assistance neighborhoods [31], in which a CheR bound to one receptor dimer can methylate six neighboring receptor dimers, whereas a CheB bound to one receptor dimer can demethylate four or five neighboring receptor dimers.

The model also represented spatially oriented processes of molecular self-assembly, where CheA and CheW could join in the cytoplasm to form new agents representing the complex, and these could assemble to fixed receptors to form CheW−CheA bridges between receptor trimers. An emergent property of this self-assembly was the rate of CheA autophosphorylation, which was dependent upon the resultant connectivity in the receptor lattice. The connectivity was in turn dependent upon the ratio of CheA to CheW used in the simulation (data not shown). Such self-assembly processes play critical roles in biological systems, and the ABM was a straightforward yet powerful way of representing such a process.

### Explicit Representation Facilitates Hypothesis and Scenario Testing

The Chemoscape model explicitly and individually represented all known chemotaxis signaling proteins along with the rules governing their behavior. This provided the ability to readily modify rules based on the variety of published experimental data from mutant bacteria expressing chemotaxis proteins with altered concentrations and/or biochemical activities. We could then compare the model's system-wide emergent behavior to that of the mutant cells expressing these altered proteins. This allowed us to rapidly test a variety of different scenarios and hypotheses (many of which are not shown for brevity). The only other chemotaxis simulation we know of with this capability is BCT [32]–[34], which represents bulk concentrations using a series of about 90 ordinary differential equations. In contrast to Chemoscape, BCT's differential equations were not spatially localized, and the system was tuned by modifying the dissociation constants.

### Parameter Definition

Our agent-based approach didn't directly solve the hard problem of model parameter selection. It is not known how sensitive to perturbation in reaction rates or protein concentrations the chemotactic system is. It may be that different parameters are similarly capable of supporting chemotaxis. For instance, it is known that the relative proportions of some chemotaxis proteins can be substantially altered without compromising precise adaptation [35], [36]. *In vivo*, the absolute concentration of chemotaxis proteins in an *E. coli* cell varies 10-fold, but the relative proportions of the signaling proteins remain constant [37]. Also, cells that simultaneously over express all chemotaxis proteins up to six-fold remain chemotactic [38]. These observations indicate that it might be more important to get the ratios of components right rather than absolute concentrations.

Because of these observations, we did not try to find a direct relationship between agent abundance on a two-dimensional grid to protein abundance in an actual cell; instead we focused on models that implemented the correct biological ratios of these components. In a discrete agent-based model, beyond a certain critical population size threshold, it is the ratios of population components that matter for emergent behaviors, not their absolute concentrations. Rather than trying to exhaustively explore this parameter space, we focused on the underlying forms of rules that govern protein interactions at the biochemical level, and their resultant emergent behavior.

The bacterial cytoplasm is a crowded environment quite unlike a buffered solution in a test tube [39], [40], and it is likely that the actual rates of macromolecular signaling reactions *in vivo* are different than the rates measured *in vitro* [41]–[43]. It has been suggested that even with large amounts of time series data, many model parameters can be poorly constrained and modelers should focus on the predictions of a model instead of parameters [44]. We found several areas in which there were little if any published data that would guide parameter choice. With Chemoscape, we were able to readily test hypothesis and settle on values that lead to emergent behavior reproducing experimental observations. While that doesn't mean we found the correct parameter values, it does indicate the likelihood that our qualitative approach - such as the number and type of rules that govern agent interactions - is on target, and can be used to explain how the complex of chemotaxis behaviors stem from the simple, local interactions that occur amongst the components.

### Biological Insights from Chemoscape

Biological insights revealed by the model include the observation that receptor cooperativity could explain the increased sensitivity of a heterogeneous receptor system to the attractant MeAsp in the presence of a second attractant Ser, and that methylation-dependent receptor coupling strength could explain the complex response curves shown *in vivo* for mixed receptor populations and varying methylation levels [16]. In addition, the model showed that in order to qualitatively reproduce the *in vivo* adaptative behavior, it is important that phospho-transfer from CheA to CheY occur at a higher rate than dephosphorylation of CheY by CheZ. Finally, the model revealed the qualitative importance of both receptor heterogeneity and methylation-dependent receptor coupling strength on providing a broad dynamic range of response for the system.

The probability of CheZ dephosphorylating CheY after they bind is quite high *in vitro* (∼99%) [45], which differs significantly from the probability used in Chemoscape for CheZ agents causing dephosphorylation of CheY agents upon encounter (20%). This discrepancy could imply that soluble agent collisions occur more frequently in the current Chemoscape model than they do *in vivo*, which then had to be offset by the lower probability setting we used for the dephosphorylation reaction.

### Related Efforts

Chemoscape is not the first attempt at modeling the experimental results of Sourjik and Berg [16], [17], which have inspired numerous theoretical analyses that seek insight into the underlying mechanisms of receptor cooperativity. One model [46] depends on poor incorporation of Tsr into mixed trimers of receptor dimers and destabilization of trimers of dimers by ligand binding, but subsequently published experimental observations [47] contradict both assumptions. Another model [48] generates results consistent with many observations by postulating a network in which CheA dimers each interact with three CheW molecules to connect trimers of receptor dimers, but is inconsistent with the ratio of CheA to CheW found in the cell [37]. Several groups created mathematical models of receptor activity that are quantitatively consistent with many experimental results. Some of these rely on the MWC model of allostery and differential equations [49]. Others use lattice receptor models and mean field approach [50], [51], but depend on weak or no interaction between Tar receptors to explain the mutant strains in Sourjik and Berg [16]. Chemoscape extends the lattice model to include multiple receptor types with a more flexible geometry than a completely filled array, as well as incorporating downstream reactions. Significantly, we found that a simple set of rules governing cooperativity in a heterogeneous receptor field could explain most if not all of the biologically observed results.

Several previous efforts share some characteristics of our agent-based model, such as StochSim, in which proteins are represented as individual software objects [21], [52]. Earlier versions of the software did not include spatial representation, but were later extended to allow for receptor arrays in discrete two-dimensional grids. A more recent software project named Smoldyn was used in models of CheY diffusion in an *E. coli* cell [53]. In these models some proteins were represented individually, but other components were modeled using differential equations (e.g. methylation/demethylation reactions in StochSim, and the receptor array in Smoldyn). Another project was Agent Cell [3], which used an agent-based model to represent individual bacteria, but did not model intracellular processes with an ABM. Our model appears to be the first that used a generalized agent-based modeling system to model all major membrane and cytoplasmic components (except the flagellar motors), providing a unified system that accounted for the known behavior of each one and demonstrated behaviors strikingly like the biological system.

### Conclusions

ABM is a relatively new modeling approach in the biological sciences, and so we should expect to discover areas of weakness or in need of refinement. Established approaches work very well for large categories of problems. Rather than present ABM in competition with these approaches at the purely quantitative level, we suggest that this promising but nascent field be appreciated for qualitative insights and hypothesis testing, with a longer term goal to match the rigor and numerical accuracy of more developed approaches. While Chemoscape has limitations due to the newness of ABM for this application, we have found many positive aspects of this approach for intracellular modeling, such as its biological intuitiveness, the rapid ability to translate hypotheses into testable models, and the straightforward representation of spatial relationships.

## Materials and Methods

All models were developed with the Ascape package, which is “a framework designed to support the development, visualization, and exploration of agent-based models” [54]. In this Java-based framework, everything is an agent. A group, or collection, of agents is contained in a scape. Rules describe the behavior of agents towards each other and with the environment. Scapes themselves are agents, and so can be part of other scapes, facilitating the building of scape hierarchies. An overview of Ascape can be found in Inchiosa and Parker [55]. The Chemoscape source code is available for download at http://bioinfo.unc.edu/Downloads/.

### Cells, Agents, and Scapes

The models consist of a virtual 2D environment in which all interactions are embedded. Models are limited to two dimensions due to the capabilities of Ascape. The environment consists of a discrete lattice of “cells” (not to be confused with biological cells) where each cell has a hexagonal geometry, representing the space inside of a bacterium. The environment is populated with agents representing proteins. A hexagonal geometry is used because it approximates certain geometrical properties of the receptor array in the biological system, such as maximal packing of cylindrical structures (e.g. receptor dimers), experimental observations of hexagonal receptor arrays in bacteria that over express receptor proteins [56], and allowing for three receptors, representing a trimer of dimers, to simultaneously contact each other in a repeating pattern.

Agents in Chemoscape, representing proteins, are differentiated into two categories, those that are soluble and free to move in the cytoplasm (foreground) and those that are membrane-embedded (part of the background). Agents in the foreground can move over the background layer, while agents in the background are not able to move. Only one agent at a time may occupy the foreground of a cell, while multiple agents may be associated with the background. Agent behavior is defined by a set of rules to model protein behaviors, such as diffusion in the cytoplasm (random movement around the scape), modification (e.g. phosphorylation) of other proteins (interaction with other agents), or autophosphorylation (self-update). A summary of the rules implemented is in Table 2. Agents interact with each other either when they are occupying the foregrounds of adjacent cells, or occupying the foreground and background of a single cell. Overviews of the model are shown in Figure S1 and Movie S1.

**Table 2:** Chemoscape rules and their effects.

Rule	Effect
Random Walk	Move around the scape randomly.
Interact	Two adjacent agents can interact with each other. Bimolecular reactions are modeled this way.
Ligand Binding	Receptors determine the fraction of time bound to ligand.
Receptor Activity State Update	Receptors decide their activity status for the next iteration, based on fraction of time ligand is bound, methylation level, and activity of neighboring receptors.
Methylate	CheR can methylate receptor agent, through Interact rule.
Demethylate	Phosphorylated CheB can demethylate a receptor in the active state.
Autophosphorylate	CheA can autophosphorylate, based on the activity of receptors it is associated with.
Phosphotransfer	Phosphorylated CheA can interact with CheY and CheB to transfer a phosphoryl group to them.
Dephosphorylate	CheZ interacts with phosphorylated CheY to dephosphorylate it.
Auto-dephosphorylate	CheB and CheY that are phosphorylated have fixed probabilities of auto-dephosphorylating each iteration.
Form Complex	Two agents can combine to form a protein complex. Specifically, CheA can combine with up to two CheWs, forming a new agent representing a CheACheW complex.
Bind	CheW can associate with a single receptor agent. When this happens, the CheW agent is removed from the lattice.

### Protein Representation

Protein types are defined in object oriented programming terms as object classes, encapsulating protein state and behavior, with specific proteins in a simulation represented as class instances (e.g. agents). We define a protein super class containing functions common to all proteins such as random walk behavior, while specific protein types are implemented as subclasses that inherit these common behaviors (Figure S2). The hierarchy allows rules for specific components of the system to be changed to test new ideas, leaving the behavior of the rest of the modules unaffected.

### Chemical Reaction Representation

Several reaction types are supported in the model, as outlined in Table 1. Unimolecular reactions are the simplest, such as stochastic autophosphorylation of CheA. For agents capable of this type of reaction, on each iteration of the simulation there is some chance that the state variable corresponding to the reaction may be changed. This is based upon the generation of a pseudo-random number in the range [0,1] from a uniform distribution and the probability of the reaction occurring. If the pseudo-random number is equal to or less then the chance of the reaction probability, than the reaction is performed and the appropriate state variables are updated.

Bimolecular reactions involve two agents, and can result in a change of state for one or both of the participating agents. For each iteration, every agent capable of interacting with other agents checks whether there is another agent in the proximity for a reaction. If there is, a pseudo-random number is generated in the same way as for unimolecular reactions to determine whether a stochastic interaction will occur. For example, phosphorylated CheA can interact with and transfer a phosphoryl group to CheY, resulting in the CheA changing to the unphosphorylated state and CheY changing to the phosphorylated state. The following example shows pseudo-code governing CheA-CheY reactions, in which a phosphoryl group is transferred from CheA to CheY. The rule is defined from the standpoint of CheA.

**if** reacting agent = CheY **then**

 **if** self is phosphorylated AND CheY is not phosphorylated **then**

  generate random number α : = [0,1]

  **if** α<reaction chance **then**

   transfer phosphoryl group from self to CheY

  **end if**

 **end if**

**end if**

Another type of reaction occurs when two agents bind together to form a protein complex, such as CheA−CheW binding. This type of reaction is similar to the bimolecular reactions, except when a stochastic interaction occurs, a rule is executed that combines the agents into a new, single agent representing the complex. Both of the interacting agents are removed from the lattice and replaced by a new complex agent representing the bound agents. In the case of CheA−CheW complexes, the new agent complex can contain either one or two CheWs, because one CheA can bind up to two CheW's.

There are also reactions in Chemoscape that cause an agent to move from the foreground, where it is mobile, to the background, where its position is fixed. This happens when a CheW agent binds to a Receptor agent. Because receptors have fixed locations in the model, when CheW binds to a receptor, it becomes fixed to the same cell location. CheA agents can also become affixed to receptor bound CheW in the following two ways. CheA in the foreground can bind to a receptor-bound CheW agent (that is not currently bound to another CheA agent) and CheA already associated with a single CheW agent can bind with another receptor-bound CheW (that is also not currently bound to another CheA agent). Free CheA agents will only move to a background cell that is adjacent to a CheW agent and also contacts two receptor agents from two distinct trimers of receptor dimers, allowing for the formation of a CheW−CheA−CheW bridge.

For modeling efficiency, we represent the small molecule receptor ligands, such as MeAsp and Ser, as bulk concentration values that are continuous across the scape, whose concentrations are able to change at each iteration. In typical experiments, a change in concentration is made, then held at a fixed value for a large number of cycles (e.g. 500 or more) to observe the system as it responds to the new concentration and stabilizes. On each iteration, the state of receptor proteins is calculated in part based upon the bulk concentration of ligand, as discussed under “Receptor Activity,” below.

### Reaction Timescales

In chemotaxis, the ligand binding reaction rates are ∼10^6^ fold higher than phosphorylation reactions [57]. If individual ligand reactions were to be represented, it would slow the model substantially. To address this, we use a model in which ligands constantly come on and off of a receptor [53], with the receptor changing its state on a slower timescale. Hence, ligand binding is represented as a time-averaged fraction of ligand occupancy of the receptor, as follows:
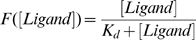
(1)Here, K_d_ depends upon receptor state, with K_d_ = 12 µM for active receptors, and K_d_ = 1.7 µM for inactive receptors, based on [58].

### Chemotaxis Model

The basic model contains agents in experimentally determined stoichiometric ratios of 3.4 receptors:2.4 CheY:1.6 CheW:1 CheA:0.5 CheZ:0.08 CheB:0.05 CheR [37]. In simulations where the methylation state of the receptors was held constant, CheB and CheR were not included. Table 2 summarizes the reaction types included in the model and the values used for their probability of occurrence on each iteration. The default scape size for simulations was 100 columns by 60 rows. For some simulations, we adjusted specific parameters as described below. The default probability values for reactions were arrived at by varying parameter values to find those that best reproduced biological observations (e.g., coupling strength that reproduced experimentally observed Hill coefficient), and/or guided trial and error for parameters that produced reasonable model behavior (e.g., CheA autophosphorylation probability, CheR/CheB methylation/demethylation rates, etc).

### Receptor Lattice

A portion of the scape was defined where receptors could be placed, termed the receptor lattice, representing the biological finding that *in vivo* receptors tend to cluster in patches on one pole of the cell [59]. The default receptor array spans 60 columns and consists of 1083 receptors. Receptor agents, representing receptor dimers, could only be placed in multiples of three, representing the biologically-determined structural unit of a trimer of dimers. Given an area where receptors were located, a predefined mask of possible trimer locations was calculated. This pattern ensured that each receptor had two intra-trimer neighbors (receptors located in adjacent lattice cells) and 2 inter-trimer neighbors (receptors separated by a single lattice cell), except on the boundary of the receptor array and in cases of a sparsely packed receptor lattice. At the start of each simulation, the receptors were laid down according to the pattern described above. The other cytoplasmic agents were then randomly placed on the scape at the start of the simulation.

This representation of a receptor lattice is similar to the receptor array in [21]. Some differences in Chemoscape are that a receptor does not have to be present at every position in the array and not all receptors have to be of the same type.

### Receptor Activity

Our model of receptor activity consists of a two-dimensional lattice of interacting receptors based on the framework presented in [21]. Receptor activity is based on the free energy difference between the active and inactive state of the receptor, and is a linear combination of four energy terms: 1) baseline energy offset, 2) ligand binding, 3) methylation level, 4) activity state of neighboring receptors. Shimizu *et al.* [21] based their ligand binding and methylation free energy parameters on experimental observations, detailed in [58], and the neighbor interaction free energy term by optimizing over the signal to noise ratio in their model. For a receptor lattice with square geometry and four interacting neighboring receptors, Shimizu *et al.* used free energy parameters of 0 kcal mol^−1^, 1.2 kcal mol^−1^, −1.2 kcal mol^−1^, and −1.9 kcal mol^−1^ for the four terms, respectively. Shimizu *et al.* also noted that at high values of interaction energy, the system behaves with an all or none “flipping” behavior. We attempted to use the same free energy parameters, but found our system started showing all-or-none behavior at a lower interaction energy value than Shimizu *et al*. The difference in models may be due to the use of hexagonal versus square lattice geometry. Also, using the formulation of Shimizu *et al.*, we noticed an edge effect on receptor activity in our model, where receptors on the edge of the lattice were more likely to be inactive due to fewer interacting neighbors. We could diminish this effect by considering the net number of active or inactive receptors, instead of the absolute number of active receptors.

Our final receptor activity equation is:
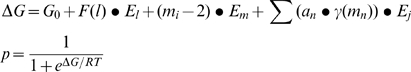
(2)where *p* is the probability that the receptor will be active in the next time step.

In initial simulations, we set G_0_ to be zero so that the activity in half methylated receptor populations is 50% of the activity of fully methylated receptor populations *in vitro* [60]. The term F(l) is from Equation 1, and incorporates the effect of time-averaged ligand binding.

The constant E_l_ determines the strength of effect of ligand binding, and was set to 2200 cal mol^−1^ for all reported simulations. This value was chosen because it resulted in most of the receptors being deactivated in simulations reproducing experimental results for our choice of *E_m_* [61]. The coefficient *E_m_* modulates the strength of the effect that methylation *m* of receptor *i* has on the activity probability, where (*m_i_*−2) is set so that at a methylation state of 2, the system is at baseline activity. *E_m_* is set to −400 cal mol^−1^, based on model performance. In Chemoscape we assume that the specific methylation sites have a small effect compared to the total number of methyl groups, so we count only the number of methyl groups per receptor [61]. The fourth term sums the contributions of the effects from cooperative interactions over all of the neighbors of receptor *n*. The coefficient E_j_ describes the strength of cooperative interactions between neighboring receptors. We attempted to base our value of *E_j_* on a parameter that would reproduce the high Hill coefficients observed by Sourjik and Berg in homogeneous over-expressed receptor populations with 2 methylations. We were unable to achieve high Hill coefficients in simulations where the baseline activity of the receptors was 50%, as is the case for a receptor population with a methylation level of 2 and G_0_ of 0. When we set G_0_ to −600 cal mol^−1^, offsetting the baseline activity, we are able to reproduce high Hill coefficients for a methylation level of 2. *a_n_* represents the activity of neighboring receptor *n*, and is set to 1, −1 for active and inactive neighbors, respectively.

The term γ(m_n_ ) allows for the strength of cooperative interactions to depend on the methylation level of neighboring receptors and was set to one for most simulations, given by:
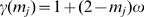
(3)where ω modulates the effect of methylation on interaction strength. ω = 0 indicates that methylation has no effect on interaction strength. In simulations where interaction strength depended on methylation level, we set ω = 0.2 so that receptors with two methyl groups contribute the normal amount to the effect of neighboring interactions. As the methylation state of the receptor increases, it has a smaller influence on its neighbors, and as the methylation state decreases, it has a larger influence on its neighbors. This mechanism has been postulated to explain certain experimental observations, including dynamic range [24] and receptor activity in mixed receptor populations [50].

To measure the Hill coefficient for receptor activity, we perform nonlinear least squares curve fitting of the model's average receptor activity to the following equation [17]:
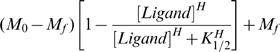
(4)where M_0_ is the pre-stimulus activity, M_f_ is the residual activity in the presence of a saturating dose of stimulus, K_1/2_ is the ligand concentration at half activity, and H is the Hill coefficient.

#### CheA activity

Because the rate of CheA autophosphorylation is several hundred times faster in the presence of CheW and receptor than in their absence [62], and because the simulations performed did not involve mutant bacteria lacking CheW or receptor, autophosphorylation of free CheA is treated as insignificant. CheA activity is therefore based on the activity of the receptors to which it was connected. The chance that an unphosphorylated CheA will phosphorylate is determined by the following equation:
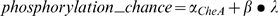
(5)where α_CheA_ represents the base chance that a CheA agent would autophosphorylate and is set to 0, β modulates the effect of being attached to a receptor, where in the active state it is set to 0.03, and λ = 0,1,2 is the number of active receptors to which the CheA agent is attached.

#### Extended pathway proteins

For extended pathway simulations, we include the following proteins that are known to be downstream from the receptors in bacteria: CheA, CheW, CheY, CheR, CheB, and CheZ. CheY and CheB have the potential to become phosphorylated when they occupy a lattice cell that contains a CheA agent in the background. CheZ has the opportunity to dephosphorylate a CheY agent when occupying an adjacent lattice cell. CheR or phosphorylated CheB have the ability to methylate or demethylate, respectively, a receptor occupying the same lattice cell.

#### Receptor activity measurements

For simulations in which receptor activity was reported for different ligand concentrations, the receptor activity was determined as follows. A datapoint containing information about the state of the system, including the number of active receptors and ligand concentration, was recorded once every 100 iterations. For each change in ligand concentration, the receptor activity of the system will adjust and then stabilize. To measure the stabilized value after each change in ligand concentration, either 700 or 1500 iterations are allowed for the system to reach stability. We then averaged the receptor activity from the next 3 or 5 data points, taken over 300 to 500 iterations. All simulations for which bulk parameters such as Hill coefficient are derived from the average of three repeated simulations, with the error bars indicating the minimum/maximum value of the three simulations. The plots from Figure 5 and Figure 6 are shown for individual (not aggregate) runs.

## Supporting Information

Figure S1 2-D random walk. Overview of the running model, with movable agents representing soluble proteins as circles, and immobile agents representing receptors as hexagons.
(5.13 MB TIF)

Figure S2 The class structure of agents in Chemoscape. At the top level is the protein agent, which implements behavior general to all protein agents, such as the ability to move stochastically and to interact with other proteins. Below that are the individual protein types. Each class implements behaviors specific to that protein type. For example, CheY can be phosphorylated, and can interact with CheA. The “receptor” type is a special subclass that is rendered immobile, and implements several subclasses for each of the major chemotaxis receptor types.
(0.03 MB PDF)

Movie S1 Chemoscape receptor activity movie.
(18.65 MB MOV)
