# Supplementary figures and images for: An Agent-Based Model of Signal Transduction in Bacterial Chemotaxis
Source: PLoS One. 2010 May 13;5(5):e9454. doi: 10.1371/journal.pone.0009454 (PMC2869346; doi:10.1371/journal.pone.0009454)

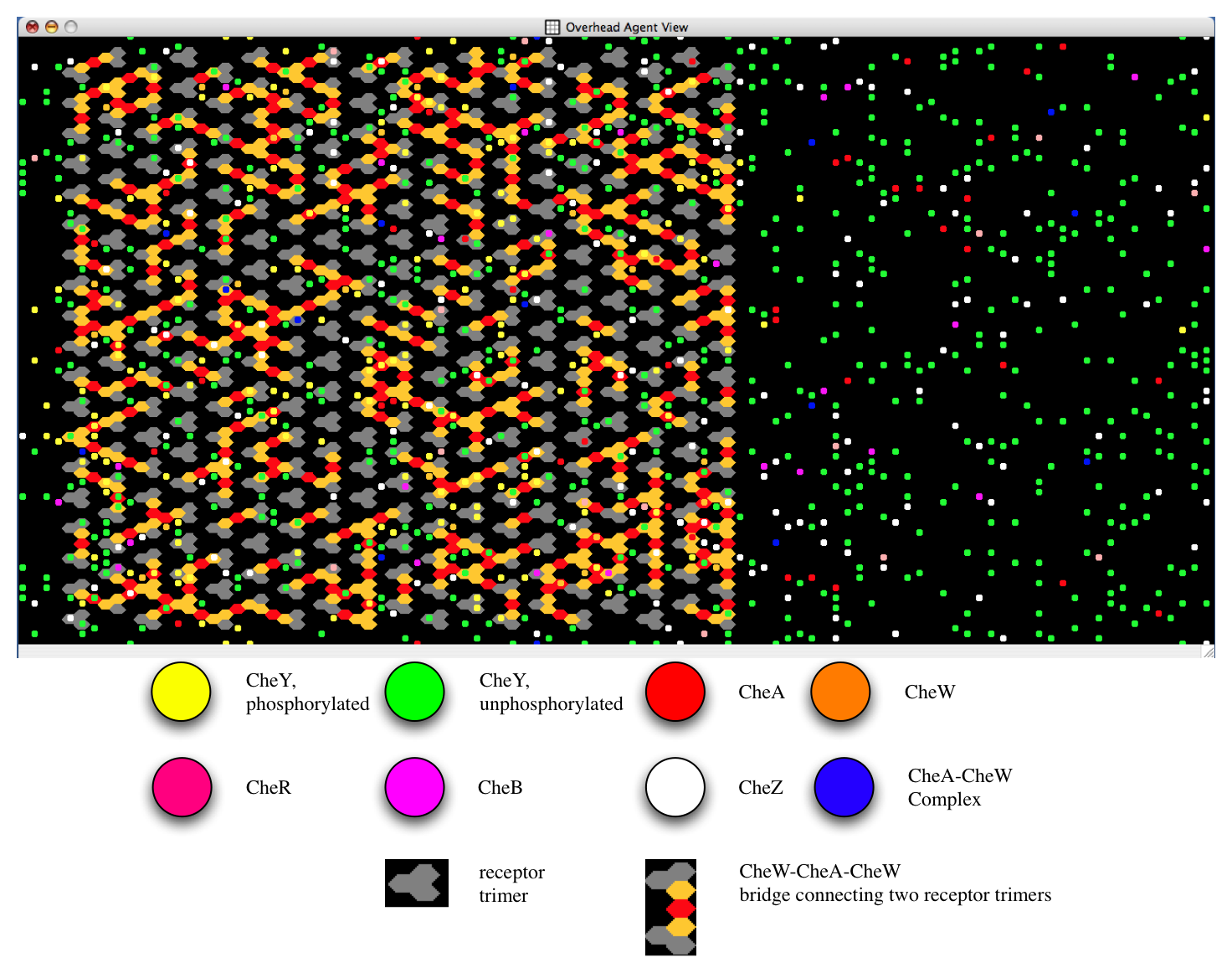

Supplement: Figure S1 — 2-D random walk. Overview of the running model, with movable agents representing soluble proteins as circles, and immobile agents representing receptors as hexagons. (5.13 MB TIF) [file pone.0009454.s001.tif]

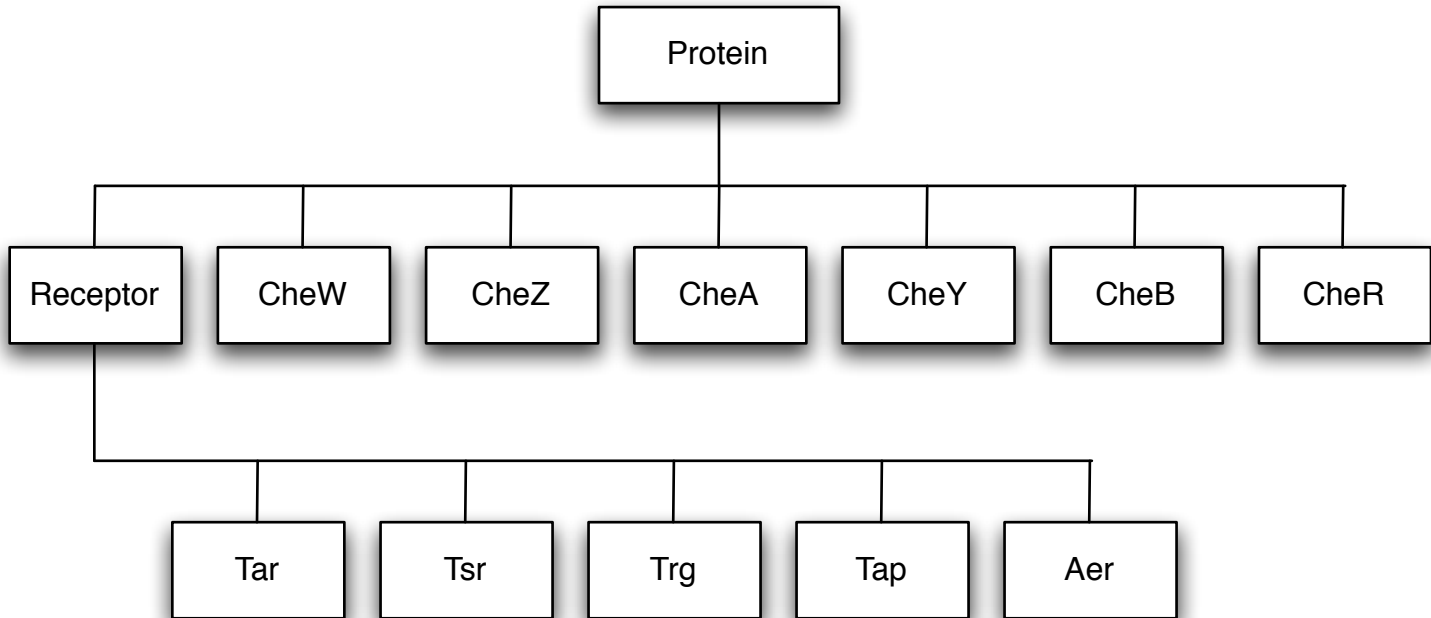

Supplement: Figure S2 — The class structure of agents in Chemoscape. At the top level is the protein agent, which implements behavior general to all protein agents, such as the ability to move stochastically and to interact with other proteins. Below that are the individual protein types. Each class implements behaviors specific to that protein type. For example, CheY can be phosphorylated, and can interact with CheA. The “receptor” type is a special subclass that is rendered immobile, and implements several subclasses for each of the major chemotaxis receptor types. (0.03 MB PDF) [file pone.0009454.s002.pdf]
